# Myelitis

**Published:** 1874-04

**Authors:** Walter Hay

**Affiliations:** No. 122 South Sangamon St.


ARTICLE II.—*Myelitis.* By WALTER HAY, M.D.

M. P.; æt. about 65; physician, in active practice many years. First seen, by request of Prof. Rea, Nov. 30, 1873. Had been visited incidentally by several eminent medical gentlemen, and prescribed casually for himself during the two months of his confinement to bed, but appears to have had no systematic treatment. Has received the impression from some of these that his case is one of locomotor ataxia; and has received, also, numerous hypodermic injections of morphia to allay pain, which, however, were unsuccessful.

Patient's mind being in an exceedingly unstable condition, it was impossible to obtain an intelligible history of the case.

Symptoms, at present, are great mental instability, resembling hysteria, characterized by alternations of outcries, groans, prayers, and imprecations, with diminution of attention, ideational incoherence, and failure of memory, expressing, in their totality, incipient senile dementia. Heart's action a little excited; arterial tension a little diminished; pulse rate, 72. Temperature normal; hygrometric condition normal. Cutaneous sensibility slightly increased over chest and abdomen, normal over all of the extremities, objectively; has "prickings of pins and needles in the soles of the feet and legs." Pain agonizing; throughout the lower extremities intermittent and lancinating; through the diaphragm and lower thoracic region continuous and intense, described as an iron band crushing his chest; involuntary respiration being somewhat impeded thereby, is supplemented by voluntary muscular action. Pain unaffected by movements of the body, which he attempts frequently, and accomplishes by means of his arms, with the assistance of an attendant.

Pupils are contracted, and tongue dry, red, and fissured.

Motility lost in the left lower extremity altogether, preserved to a slight degree in the right in adductors of thigh, in upper extremities unimpaired.

Bladder and rectum paralyzed; sphincters normal; urine dribbling constantly.

Special sensation unimpaired; pupils contracted; conjunctivæ injected; reflex irritability in lower extremities increased.

Removed, by means of the catheter, about a quart of highly alkaline urine, loaded with ammoniacal phosphates; no attempt having been made to evacuate the bladder previously. Ordered—

| | | |
|---|---|---|
| Potassii Bromid., | - - - - - - - - | grs. xv. |
| Aquæ Destil., | - - - - - - - - - - | oz. ss. M. |

every four (4) hours, and atropiæ gr. $\frac{1}{60}$ every eight (8) hours.

Dec. 1. Had passed the night as usual, suffering frightfully. Bowels evacuated, the first time in ten days. Removed about twenty ounces from the bladder. Ordered—

| | | |
|---|---|---|
| Potassii Bromid., | - - - - - - - - | grs. xxx. |
| Aquæ Destil., | - - - - - - - - | oz. i. M. |

every four (4) hours; atropiæ granules, gr. $\frac{1}{60}$ every four (4) hours; beef essence and milk *ad lib.*; occasionally a little sour ale to quench his thirst.

9 P. M. Removed similar quantity of urine. Continue treatment. Pain had ceased, except in left sciatic nerve, and respiration was freer.

Dec. 2, 9 A. M. Found patient narcotized; at 4 A. M. had been seized with hiccough, and had called in a neighboring practitioner, who had "passed a catheter, but found no urine in the bladder, and had given pulv. opii, gr. j; gum camphor, gr. ij, in two pills."

Upon inserting catheter was more fortunate than the previous operator, finding about a pint of ammoniacal urine. Ordered treatment continued, and requested consultation with Profs. Allen and Gunn, who, at 4.30 P. M., found narcosis continuing, and were convinced that a much larger quantity of opium than the alleged "one grain" had been taken.

These gentlemen, after careful examination of the case, sustained my diagnosis of chronic myelitis in its later stages, but, considering it essentially hopeless, made no suggestions for treatment.

As there were no evidences of pain, reduced doses of medicine to one-half, as before.

Death ensued Dec. 7, nothing noteworthy occurring in the meantime.

The only point of interest in the case being its diagnosis—the prognosis being hopeless—it is published simply because it has already been reported as a case of locomotor ataxia.

The diagnosis rests upon the tripod: paraplegia, without anæsthesia; cramp—painful spasm—of the diaphragm, indicated by sense of constriction around lower portion of thorax; and alkalinity of urine.

No. 122 South Sangamon St.

